# Absorbable sutures reduce pain without compromising wound healing in open clinic hand surgery

**DOI:** 10.1186/s13018-026-07159-7

**Published:** 2026-08-22

**Authors:** Mirjam Hägg, Cecilia Mellstrand Navarro, Johanna von Kieseritzky

**Affiliations:** 1 Department of Hand Surgery, Södersjukhuset, Stockholm, Sweden; 2 Department of Clinical Research and Education, Södersjukhuset, Karolinska Institutet, Stockholm, Sweden; 3 Department of Clinical Sciences, Danderyd Hospital, Section for Orthopaedics, Karolinska Institutet, Stockholm, Sweden

**Keywords:** Absorbable sutures, Non absorbable sutures, Hand surgery, Postoperative infection, Infection surgical wound, Wound rupture

## Abstract

**Background:**

This prospective cohort study aimed to compare absorbable and non-absorbable sutures in the context of open clinic hand surgery, focusing on postoperative wound infection, dehiscence, patient-reported pain during dressing or suture removal, and healthcare time utilization during follow-up.

**Methods:**

A total of 270 adult patients undergoing minor hand procedures in an outpatient surgical setting were enrolled. Patients were alternately assigned, on a biweekly basis, to receive either 5-0 polyglactin 910 (Vicryl Rapid®, Ethicon®) or 5-0 monofilament nylon (Ethilon®, Ethicon®) for skin closure. Wounds were assessed at a standardized two-week postoperative follow-up by trained nursing staff. The presence of wound infection or dehiscence was documented. The time required for inspection and dressing, or suture removal was recorded in minutes. Pain during dressing or suture removal was evaluated by patients using a visual analog scale (VAS).

**Results:**

At follow-up, no significant difference was observed in infection rates between the two groups, with one case in each group requiring antibiotic treatment (*p* = 1.0). Superficial wound dehiscence occurred in 13 patients in the absorbable suture group and 11 in the non-absorbable group (*p* = 0.390), with no cases requiring reoperation. Median time spent on wound care during the follow-up visit was five minutes in both groups. However, patients in the absorbable suture group reported significantly lower pain scores on the VAS compared to those with non-absorbable sutures (*p* < 0.001).

**Conclusion:**

Absorbable sutures offer a safe alternative to non-absorbable sutures in outpatient hand surgery, providing equivalent outcomes in terms of wound healing while significantly reducing discomfort during follow-up care. These findings support the use of absorbable sutures as a patient-friendly option in ambulatory hand surgical practice.

*Trial registration number* The study was pre-registered on ClinicalTrials.gov 2021-02-26. (identifier: NCT04812275).

**Level of evidence:**

II.

**Supplementary Information:**

The online version contains supplementary material available at https://doi.org/10.1186/s13018-026-07159-7.

## Introduction

Achieving optimal wound closure is essential in surgical procedures to promote efficient healing and ensure aesthetically satisfactory results, and sutures remain the most commonly employed method for this purpose [1–4]. In the field of hand surgery, non-absorbable sutures are the conventional choice, largely due to concerns that absorbable alternatives may trigger immunogenic responses during the healing process. Such reactions have historically been linked to complications in wound healing and suboptimal cosmetic outcomes [5, 6]. A notable exception to this standard exists in pediatric hand surgery, where minimizing patient discomfort by eliminating the need for suture removal has been prioritized over potential inflammatory reactions [7, 8].

Although several studies have examined the performance of various suture materials in elective hand surgery, many suffer from notable methodological limitations [9]. These include small sample sizes, often involving 50 patients or fewer [10–13], or narrow focus on a single diagnosis or procedure [14–17]. Furthermore, the reported outcomes vary widely across the literature—from general patient satisfaction and scar aesthetics to postoperative pain, scar sensitivity, pillar pain, inflammation, and granuloma formation—complicating efforts to synthesize results or perform meta-analyses [11, 13, 16, 18–21]. In some cases, the studies also lack robust evidence levels or evaluate suture materials that are not widely available in global clinical practice [22, 23]. Only one previous study has addressed outcomes from the use of Nylon versus Vicryl in the treatment of acute hand lacerations [24].

In light of these gaps, the present study aimed to conduct a prospective comparative cohort trial assessing absorbable and non-absorbable sutures in both elective and acute hand surgery procedures performed in an outpatient setting. The primary endpoint was the incidence of postoperative wound infection. Secondary outcomes included wound dehiscence, time required for follow-up wound care, and patient-reported pain during dressing and/or suture removal.

## Methods

This prospective, comparative cohort study included 270 adult patients who underwent hand surgery in an outpatient operating unit at the Department of Hand Surgery between April 2021 and March 2023. Ethical approval was granted by the Swedish Ethical Review Authority (DNR 2020-06004), and the study was pre-registered on ClinicalTrials.gov (identifier: NCT04812275).

### Eligibility and enrollment

All patients were screened for inclusion on the day of surgery, and written informed consent was obtained prior to participation. Eligible participants were individuals aged 18 years or older undergoing elective or emergency hand surgery under local anesthesia in a day-surgery setting. Exclusion criteria comprised patients under the age of 18, those with burn injuries, a history of impaired wound healing or keloid formation, ongoing systemic corticosteroid treatment, procedures involving skin grafts, active infections, or traumatic injuries with an elevated risk of infection. Specifically, wounds showing early signs of infection, visible contamination, or open fractures were excluded. Clean incised wounds without contamination were considered eligible.

### Study design and allocation

Patients were allocated to one of two intervention groups based on the calendar week of their surgery. A quasi-randomization approach was used: surgeries performed during weeks with odd numbers received absorbable sutures (5-0 polyglactin 910, Vicryl Rapid®, Ethicon®), while procedures conducted during even-numbered weeks were closed using non-absorbable sutures (5-0 monofilament nylon, Ethilon®, Ethicon®).

### Surgical technique and operators

All procedures were performed under local anesthesia by the attending surgeon assigned to the outpatient surgical unit on that day. A total of 23 surgeons, rotating through the clinic on a scheduled basis, participated in the study. Surgeons were instructed to close the skin using cutaneous sutures, with the choice of suture technique—interrupted, interrupted mattress, or continuous—left to their discretion. No subcuticular or intracutaneous suturing was employed.

### Follow-up and outcome assessment

At two weeks postoperatively, patients returned for follow-up conducted by an independent, unblinded registered or assistant nurse. During this visit, nurses documented the presence of wound infection and dehiscence as binary outcomes. Additionally, they recorded the time (in whole minutes, using a standard analog wall clock) required for wound inspection, dressing change, and, where applicable, suture removal.

For patients with absorbable sutures, wounds were cleansed using soap and a sponge. Remaining suture knots were assessed and removed only if they detached without resistance; otherwise, patients were instructed to continue gentle cleansing at home to facilitate natural suture dissolution. Nurses identified infection based on clinical signs, including erythema, localized swelling, increased warmth, and purulent discharge. Wound dehiscence was assessed visually, defined as any clearly visible separation of wound edges.

### Pain and clinical judgment

Patients were asked to report discomfort associated with dressing and/or suture removal using a visual analogue scale (VAS). In cases where wound rupture or signs of infection were observed, a senior hand surgeon evaluated the patient to determine whether medical intervention—such as antibiotics or surgical debridement—was warranted.

### Data management

Demographic information, diagnoses, and all clinical outcomes were recorded in pseudonymized form and analyzed at the group level to maintain confidentiality.

## Statistics

Prior to the initiation of the study, a sample size calculation was conducted. Based on an expected infection rate of 3%, it was estimated that a sample of 114 patients per group would be needed to detect a 10% difference in infection rates with a statistical power of 80%. To account for an anticipated dropout rate of 15%, the total number of participants was increased to 270.

Continuous variables with ordinal or non-normally distributed data were reported as medians alongside their interquartile ranges (IQR). For completeness, means and standard deviations (SD) were also provided. Comparisons between groups for continuous variables were conducted using the Mann–Whitney U test.

Categorical variables were summarized as frequencies and percentages. Group differences in categorical outcomes were assessed using the Chi-square test. In cases where expected cell counts were low, Fisher’s exact test was applied instead.

In addition, a post hoc binary logistic regression analysis was performed for the outcomes “infection” and “wound rupture.” The model adjusted for potential confounders, including suture type (group allocation), smoking status, and the presence of diabetes. Results were presented as odds ratios (OR) with corresponding 95% confidence intervals (CI).

## Results

**Fig. 1:**
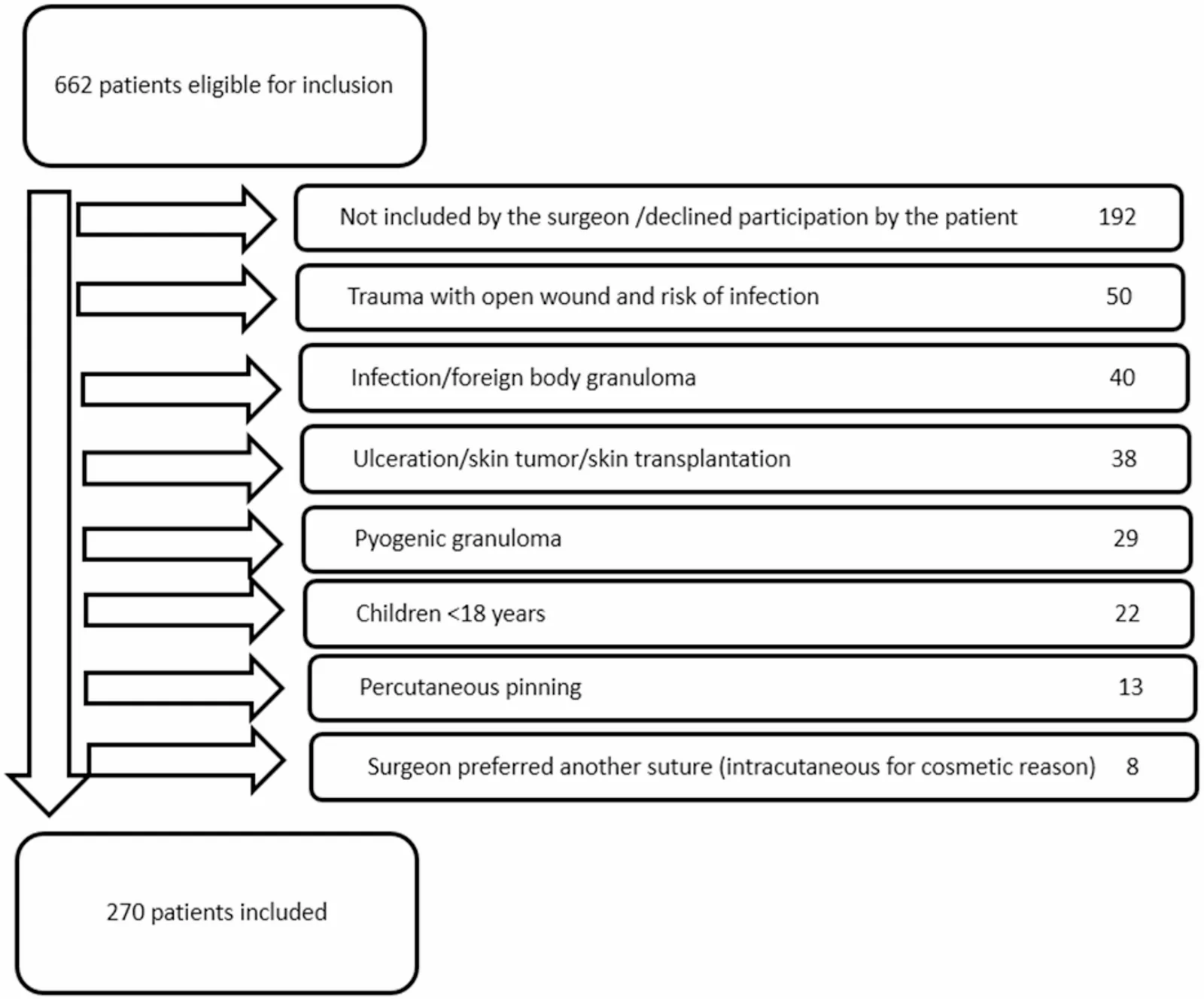
Inclusion

A total of 662 individuals were assessed for eligibility during the study period (see Fig. 1 for participant flow). Of these, 270 patients met the inclusion criteria and were enrolled in the study. Among them, 150 were assigned to the non-absorbable suture group, and 120 to the absorbable suture group. The primary outcome was successfully evaluated in 268 participants.

Baseline demographic data are summarized in Table 1, and diagnostic categories are outlined in Table 2. No statistically significant differences were observed between the two groups with respect to demographic variables or preoperative characteristics.

**Table 1:** Study population demographics in a comparative cohort trial of suture materials used in outpatient hand surgery

	Absorbable sutures *n* = 120		Non-absorbable sutures *n* = 150		*p*-value
	*n*		*n*		
Females	75	63%	90	60%	0.707*
Smokers	15	13%	16	11%	0.658**
Diabetes	10	8%	23	15%	0.093*
Surgery to dominant hand	71	59%	71	49%	0.137*

	mean (SD)	Median (IQR)	mean (SD)	Median (IQR)	
Age at inclusion	58 (16)	*57 (22)*	56 (16)	*58 (22)*	0.628***

n, numbers

*Fisher’s exact test

**Chi^2^

*** Mann Whitney U-test

SD, Standard deviation

IQR, Interquartile range

**Table 2:** Diagnoses represented in a prospective cohort study evaluating suture materials in outpatient hand surgery

	*n*
Carpal tunnel syndrome	129
Trigger finger	55
Extraction of osteosynthesis	24
Skin tumors	23
Joint conditions (ganglion, osteoarthritis etc.)	19
Foreign body granuloma without skin engagement	6
De Quervain	5
Tendon injury	3
Nerve conditions (not compressions)	2
UCL injury	2
Revision of amputation stump	1
Fracture	1
Total	270

At the two-week postoperative evaluation, no statistically significant differences were observed between the absorbable and non-absorbable suture groups regarding wound infection or wound rupture (Table 3). One patient in each group exhibited clinical signs of infection (*p* = 1.0), and both cases were managed with antibiotic therapy. One had a trigger release and the other a skin tumor removal. Superficial wound dehiscence was noted in 13 patients in the absorbable suture group and in 11 patients in the non-absorbable group, with no significant difference between groups (*p* = 0.390) (Table 3). None of the patients required surgical re-intervention.

A binary logistic regression analysis was performed to evaluate factors potentially associated with the primary outcome, wound infection. After adjusting for diabetes, smoking status, and suture type, no statistically significant associations were identified. The presence of diabetes yielded an odds ratio (OR) of 7.890 (95% CI 0.454–137, *p* = 0.156), while smoking was associated with an OR of 7.346 (95% CI 0.423–127, *p* = 0.171). The type of suture used had no significant impact on infection risk (OR = 0.733, 95% CI 0.041–12.967, *p* = 0.832).

In contrast, diabetes showed a significant association with wound rupture in the adjusted model (OR = 3.131, 95% CI 1.105–8.869, *p* = 0.032). However, smoking (OR = 2.088, 95% CI 0.704–6.187, *p* = 0.184) and suture type (OR = 0.599, 95% CI 0.251–1.429, *p* = 0.248) were not significantly related to wound dehiscence.

The time required for wound inspection, dressing removal, and/or suture removal during the two-week follow-up was comparable between the groups, with no statistically significant differences observed. However, patient-reported discomfort, assessed using the visual analogue scale (VAS), was significantly lower in the absorbable suture group (*p* < 0.001), as shown in Table 3.

**Fig. 2:**
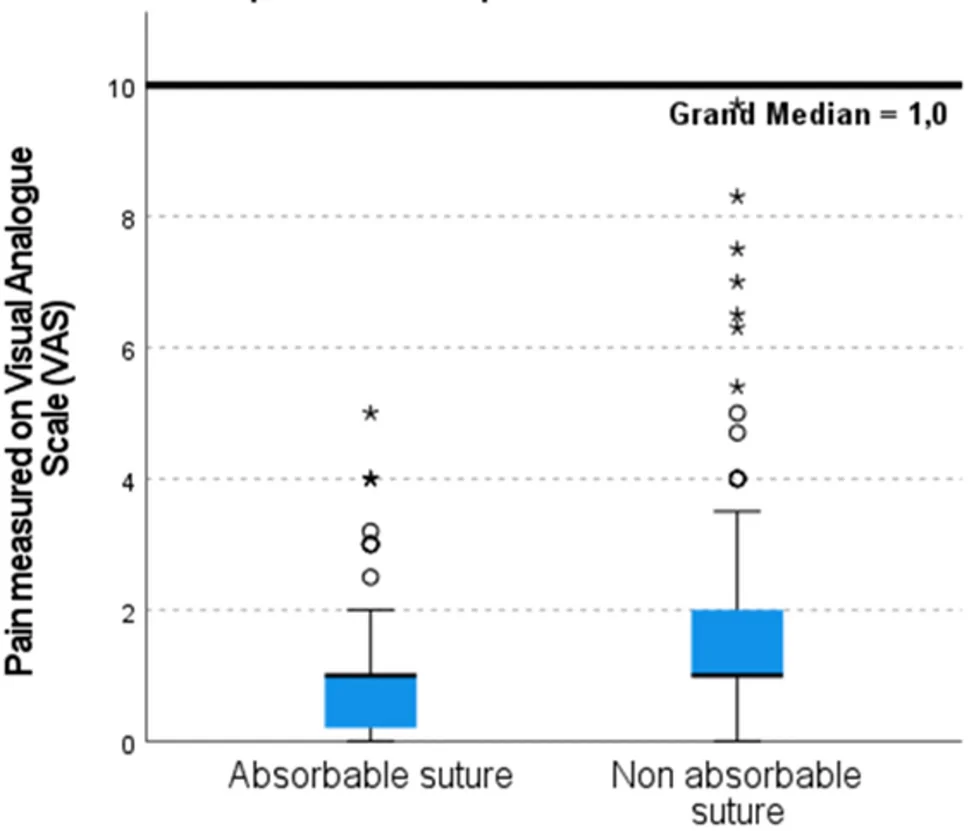
Distribution of pain at dressing and suture removal as reported on the visual analog scale

While the median VAS scores between the two groups were identical, the overall distribution of pain scores varied substantially, as illustrated in Fig. 2.

**Table 3:** Postoperative results from a prospective cohort trial comparing suture types in outpatient hand surgery

	Absorbable sutures *n* = 119		Non-absorbable sutures *n* = 149		*p*-value
	*n*		*n*		
Wound infection	1	0.8%	1	0.7%	1.0*
Wound rupture	13^#^	11%	11^¤^	7%	0.390*

	mean (SD)	median (IQR)	mean (SD)	median (IQR)	
Time spent in minutes for removal of dressing or dressing and sutures	5.0 (4.0)	*5.0 (2)*	5.9 (5.2)	*5.0 (2)*	0.141**
Pain (VAS) at removal of dressing or dressing and sutures	0.97 (1.0)	*1.00 (1)*	1.67 (1.7)	*1.00 (1)*	< 0.001** < 0.001***

n, numbers

*Fisher’s exact test

** Mann Whitney U-test

*** Student’s* t*-test

SD, Standard deviation

IQR, interquartile range

VAS, Visual Analogue Scale

^#^ 8 carpal tunnel syndromes, 4 trigger fingers, 1 joint condition

^¤^ 8 carpal tunnel syndromes, 1 trigger finger, 2 skin tumors

## Discussion

This prospective, comparative cohort trial involving 270 patients evaluated the outcomes of absorbable versus non-absorbable sutures in minor hand surgery performed under local anesthesia in an outpatient clinic. The findings demonstrated no significant difference between the two groups with respect to local wound infection or superficial wound dehiscence.

These results align with prior work by Shetty et al. who similarly reported no increased infection risk with either suture type in the context of acute lacerations [24]. Additional smaller studies have also documented low infection rates regardless of whether absorbable or non-absorbable sutures were used [10, 12, 15, 18]. However, our study contributes new insight into patient experience, as we observed significantly greater pain during dressing or suture removal in the non-absorbable group. This finding is supported by earlier research: Theopold et al. noted reduced pain with Vicryl compared to Novafil in carpal tunnel release surgery [12], and Erel et al. reported a similar benefit when comparing Vicryl to Prolene [15]. Leary et al. found no difference between prolene and Vicyl Rapide in a randomized controlled trial [17].

The absorbable suture material used in this study, 5-0 polyglactin 910 (Vicryl Rapid®, Ethicon®), is designed to degrade quickly. Despite its rapid loss of tensile strength, it appears sufficient for closing small incisions in hand surgery. Its fast resorption offers a clear advantage in terms of reducing pain during postoperative care, particularly at the time of dressing removal.

Wade et al. [9], in a 2018 Cochrane review, analyzed five randomized trials assessing absorbable versus non-absorbable sutures in carpal tunnel surgery, encompassing a total of 255 patients [11, 12, 14, 15, 18]. The authors identified multiple limitations within these studies, including inadequate randomization procedures, absence of blinding, small sample sizes, and restricted generalizability. In contrast, our study enrolled a larger cohort, included a broader range of diagnoses (including some acute cases), and involved all surgeons within our department, improving the representativeness of the results. Additionally, group allocation was clearly defined, and the postoperative assessments at two weeks were conducted independently of the operating surgeon, further minimizing potential bias.

Although economic evaluation was not a primary endpoint, there are relevant financial considerations. While the cost of absorbable sutures used in this study was approximately double that of non-absorbable ones, the potential for reducing postoperative clinic visits—particularly for suture removal—may offer overall savings to the healthcare system. Patients using absorbable sutures were able to perform much of the wound care themselves, possibly eliminating the need for nurse-led follow-up, and avoiding time off work or other personal inconveniences. Given the comparable safety profiles observed in terms of infection and wound healing, these practical benefits support the broader implementation of absorbable sutures in outpatient hand surgery. However, patient-reported outcomes beyond pain—such as satisfaction, scar quality, or psychological burden—were not evaluated and may be worthwhile subjects for future research.

The principal strength of this investigation lies in its pragmatic design, substantial sample size, and inclusion of a diverse patient population representing various surgical indications. The quasi-randomized allocation strategy helped to reduce selection bias while maintaining real-world clinical applicability.

Nonetheless, several limitations should be acknowledged. The relatively short follow-up period (two weeks) restricts the ability to assess long-term wound healing, scar formation, or patient satisfaction. Moreover, the quasi-randomized design lacks the methodological rigor of a blinded RCT. Patient inclusion was slower than anticipated, potentially influenced by the COVID-19 pandemic, which may have led some patients to decline participation to avoid additional clinic visits. Unfortunately, reasons for declining enrollment were not systematically documented, limiting insight into potential selection bias.

## Conclusion

In this prospective comparative cohort study, the use of absorbable and non-absorbable sutures in outpatient hand surgery was associated with comparable rates of wound infection and dehiscence. Both materials appear clinically safe. However, absorbable sutures were associated with significantly less pain during postoperative care and may reduce the need for follow-up visits. Based on these findings, absorbable sutures represent a preferable choice in open clinic hand surgery, offering both clinical and practical benefits without compromising safety.

## Supplementary Information

Below is the link to the electronic supplementary material.

Supplementary Material 1.

## Data Availability

The datasets used and/or analyzed during the current study are available in a supplementary file added in this Resubmission.
